# Neutron Exposures in Human Cells: Bystander Effect and Relative Biological Effectiveness

**DOI:** 10.1371/journal.pone.0098947

**Published:** 2014-06-04

**Authors:** Isheeta Seth, Jeffrey L. Schwartz, Robert D. Stewart, Robert Emery, Michael C. Joiner, James D. Tucker

**Affiliations:** 1 Department of Biological Sciences, Wayne State University, Detroit, Michigan, United States of America; 2 Department of Radiation Oncology, School of Medicine, University of Washington, Seattle, Washington, United States of America; 3 Department of Radiation Oncology, Wayne State University, Detroit, Michigan, United States of America; ENEA, Italy

## Abstract

Bystander effects have been observed repeatedly in mammalian cells following photon and alpha particle irradiation. However, few studies have been performed to investigate bystander effects arising from neutron irradiation. Here we asked whether neutrons also induce a bystander effect in two normal human lymphoblastoid cell lines. These cells were exposed to fast neutrons produced by targeting a near-monoenergetic 50.5 MeV proton beam at a Be target (17 MeV average neutron energy), and irradiated-cell conditioned media (ICCM) was transferred to unirradiated cells. The cytokinesis-block micronucleus assay was used to quantify genetic damage in radiation-naïve cells exposed to ICCM from cultures that received 0 (control), 0.5, 1, 1.5, 2, 3 or 4 Gy neutrons. Cells grown in ICCM from irradiated cells showed no significant increase in the frequencies of micronuclei or nucleoplasmic bridges compared to cells grown in ICCM from sham irradiated cells for either cell line. However, the neutron beam has a photon dose-contamination of 5%, which may modulate a neutron-induced bystander effect. To determine whether these low doses of contaminating photons can induce a bystander effect, cells were irradiated with cobalt-60 at doses equivalent to the percent contamination for each neutron dose. No significant increase in the frequencies of micronuclei or bridges was observed at these doses of photons for either cell line when cultured in ICCM. As expected, high doses of photons induced a clear bystander effect in both cell lines for micronuclei and bridges (p<0.0001). These data indicate that neutrons do not induce a bystander effect in these cells. Finally, neutrons had a relative biological effectiveness of 2.0±0.13 for micronuclei and 5.8±2.9 for bridges compared to cobalt-60. These results may be relevant to radiation therapy with fast neutrons and for regulatory agencies setting standards for neutron radiation protection and safety.

## Introduction

Ionizing radiation leads to chromosome damage of the type seen in cancer cells. Ionizing radiation is also an effective method for treating tumors because it can be localized to the tumor and is a potent inducer of DNA double-strand breaks, a highly toxic form of DNA damage. While much has been learned about x-ray and gamma-ray effects on cells and whole organisms, less is known about the biological effects of neutrons. Neutrons are highly energetic uncharged particles that induce more severe DNA damage than photons and are therefore more effective than photons in controlling radioresistant tumors. The relative biological effectiveness (RBE) of neutrons has been reported to be as low as 1 and perhaps higher than 10 depending on the tissue type, neutron energy and the biological endpoint being measured [1]. Neutrons were listed as a carcinogen for the first time in the Eleventh Report on Carcinogens [2]. High levels of neutron irradiation occur in patients receiving neutron therapy, while low levels of neutron exposure occur in patients treated with high energy photons and protons. Other sources of low level neutron irradiation may include occupational exposure to workers at nuclear power plants and accelerator facilities, astronauts, airline crews and passengers on high altitude flights [3]–[14], as well as radiation incidents such as the Hiroshima-Nagasaki atomic bomb explosions and the tsunami-induced radiation leak at the Fukushima Daiichi site in Japan [15].

One of the major paradigm shifts in the field of radiation biology was the discovery of non-targeted effects such as the bystander effect in which cells in the vicinity of radiation-damaged cells behave as though they were also irradiated [16]–[20]. In addition, late effects such as chromosomal instability may increase susceptibility to cancer [21]. Thus, cells that are directly damaged are not the sole targets of radiation exposure. Cells that do not absorb radiation directly may nevertheless be damaged or altered in ways that do not become apparent for many cell generations. Such non-targeted effects may have serious implications for human health and may cause cancer. Therefore, the risks of ionizing radiation need to be analyzed in terms of both direct and non-targeted effects.

The bystander effect has been observed repeatedly in mammalian cell lines, including human skin fibroblasts, epithelial cells and leukemic cells in response to ionizing photons [17], [22]–[36]. Depending upon the cell and tissue type, bystander signals can be transmitted either through the culture medium [17] or by cell-to-cell contact including gap junctional communication [37]. Some of the candidate intercellular signaling molecules that have been implicated in bystander effects are reactive oxygen species [20], [38], reactive nitrogen species [20], [38], nitric oxide [27], [38], cytokines such as TGFβ and interleukin 8 [39], and small molecules such as amino acids [37], [40], [41]. The involvement of intracellular signaling molecules including mitogen-activated protein kinases (MAPK) and their downstream proteins [42], [43], protein kinase C (PKC) isoforms [44], tumor protein 53 (p53) [45], [46], cyclin-dependent kinase inhibitor 1A (CDKN1A, p21) [47], ataxia telangiectasia mutated protein (ATM) [44], and ataxia telangiectasia and Rad3 related (ATR) DNA-dependent protein kinase (DNA-PK) [44], [48] have also been implicated. Recently, some laboratories have suggested that the presence of serotonin in the serum is one of the key factors involved in the bystander effect [49]–[51], however this finding has been disputed [52].

Most bystander effect studies have been performed using x-rays [22], [24], [29], gamma rays [17], [35], [53] and alpha particles [47], [54], [55], however, little has been done concerning the effects of neutron radiation [56]. Such information might be important for risk estimation in response to neutron exposure. No conclusive cytogenetic evidence exists to support or refute the existence of non-targeted effects in cellular responses to neutrons. A bystander effect following neutron exposure has been observed in Chinese hamster ovary cells [57], but no effect was seen in zebrafish irradiated *in vivo* [56]. There are no available cytogenetic data concerning the bystander effect in human cells in response to neutrons.

Here we used the cytokinesis-block micronucleus assay to address the question whether neutrons induce a bystander effect in normal human lymphoblastoid cell lines. We also assessed the RBE of fast neutrons produced by 50.5 MeV protons incident on a Be target (∼17 MeV average neutron energy) compared to cobalt-60 gamma radiation. For the endpoints of micronuclei and nucleoplasmic bridges, we found no evidence to indicate that neutrons induce a bystander effect in normal human lymphoblastoid cells. The measurements indicate that the neutron RBE for directly damaged cells compared to cobalt-60 gamma rays is 2.0±0.13 for micronuclei and 5.8±2.9 for bridges.

## Results

### Nuclear Division Indices

The Nuclear Division Indices (NDI) for all experimental conditions were high enough to enumerate micronuclei and bridges, with the exception of cells irradiated with the highest two neutron doses (3 Gy and 4 Gy). Here, radiation-induced cell cycle delays precluded obtaining sufficient numbers of scoreable binucleated cells (Table 1). Although these cells had NDI values similar to the 3 Gy and 4 Gy photon-irradiated cells, they could not be scored because their morphology was not compatible with accurate damage assessments. NDI's for the two replicate neutron bystander experiments were very similar (p>0.05).

**Table 1:** Average number of nuclei per cell in directly exposed and bystander cells for neutron and cobalt-60 gamma rays.

Radiation dose (Gy)	Nuclear division index			
	GM15510 cells		GM15036 cells	
	Direct	Bystande^a^	Direct	Bystande^a^
Neutrons				
0 (pooled)^b^	2.27	2.11	1.98	2.11
4 (media only)^c^	-	2.23	-	2.09
0.5	1.61	2.20	1.39	2.00
1	1.46	2.06	1.40	1.98
1.5	1.35	1.90	1.25	2.33
2	1.24	2.18	1.18	1.93
3	1.20^d^	2.07	1.14^d^	2.13
4	1.11^d^	2.30	1.09^d^	2.14
Cobalt-60 γ				
0	1.92	1.79	1.79	1.71
4 (media only)^c^	-	1.97	-	1.83
0.5	1.87	1.89	1.58	1.79
1	1.64	1.92	1.51	1.93
2	1.24	1.82	1.21	1.81
3	1.13	1.92	1.19	1.82
4	1.06	1.90	1.13	1.85

a Neutron data shown are for replicate 1; the values for replicate 2 were similar (p>0.05).

b Combined values of the controls (pre-radiation, post-radiation and transportation control).

c Media without cells was irradiated with 4 Gy and transferred to unirradiated cells.

d Too few high quality binucleated cells were available for scoring.

### Direct damage induced by neutrons

**Figure 1:**
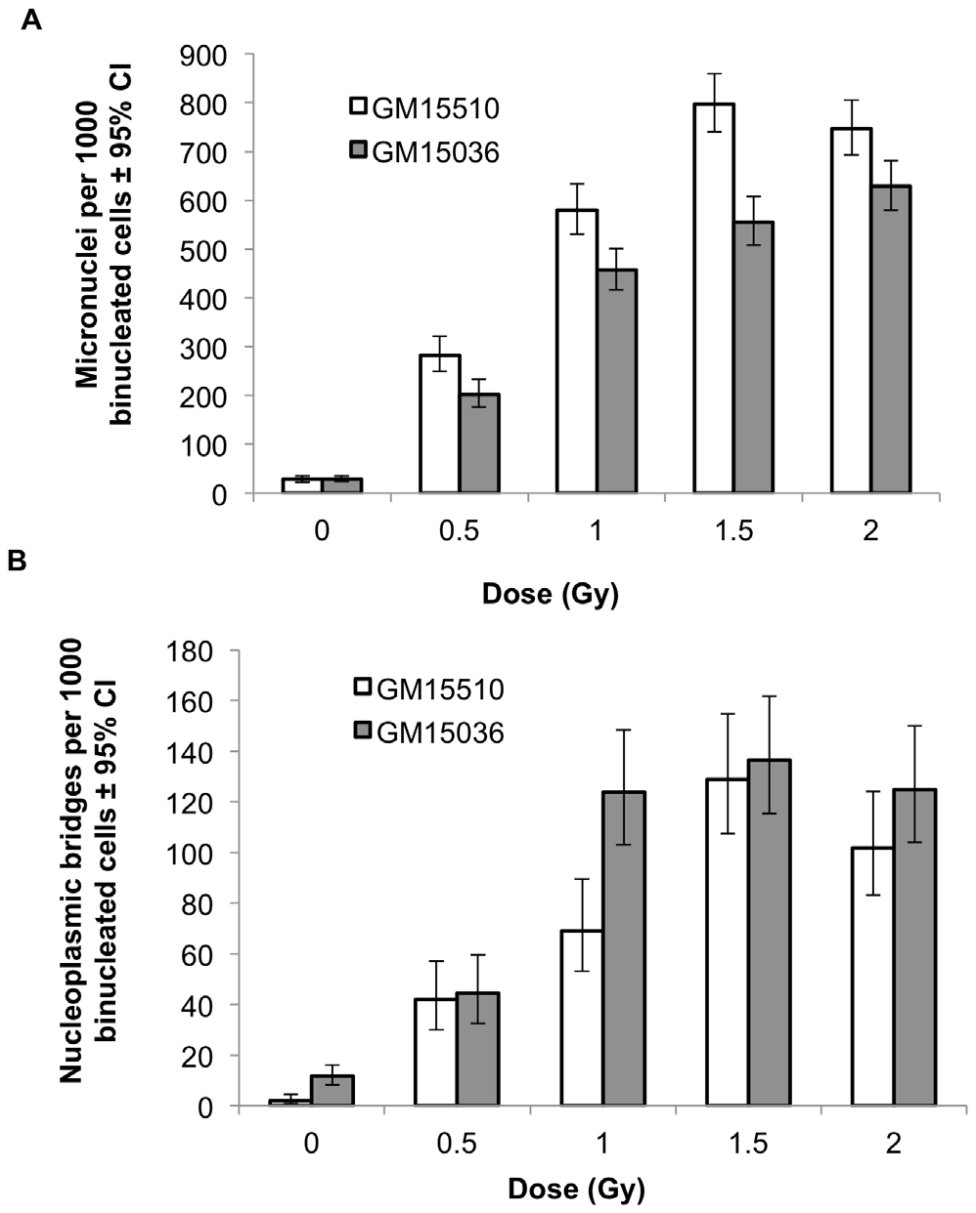
Micronuclei (a) and nucleoplasmic bridges (b) per 1000 binucleated cells in normal human lymphoblastoid cells directly irradiated with neutrons. Vertical lines represent the 95% confidence intervals.

For both cell lines, direct irradiation with neutrons resulted in clear dose-responsive increases in the number of micronuclei (Figure 1a) and nucleoplasmic bridges (Figure 1b) per 1000 binucleated cells. Since the three 0-dose controls (i.e., pre-radiation, post-radiation, and transportation control) for micronuclei and for nucleoplasmic bridges were not statistically different from each other as determined by ANOVA, we report only the pooled control values for these endpoints.

### Lack of bystander effect in response to neutrons

**Figure 2:**
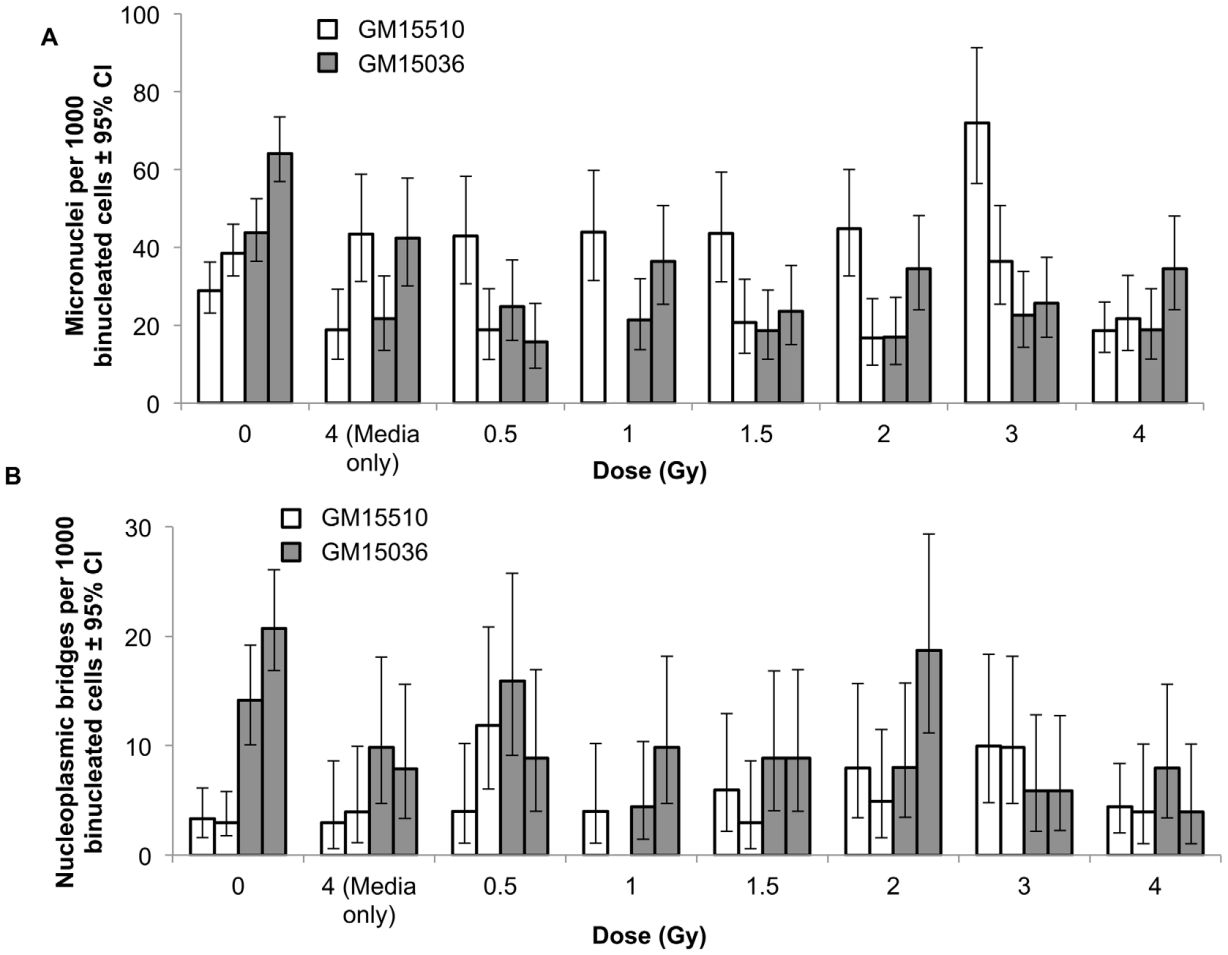
Micronuclei (a) and nucleoplasmic bridges (b) per 1000 binucleated cells in normal human lymphoblastoid cells cultured in conditioned media from neutron irradiated cells. For each treatment (i.e., cell line and dose group) the left-hand bar in each pair is replicate 1 and the right-hand bar is replicate 2. The 1 Gy data are missing for GM15510 cells replicate 2 because the sample was lost. Vertical lines represent the 95% confidence intervals.

Cells treated with ICCM from neutron irradiated cells did not show significant increases in micronuclei frequencies compared to sham treated controls for either replicate experiment and for either cell line (Figure 2). As expected, the media-only control and the 0 Gy control were not statistically different (p>0.05). For GM15510 cells, although the micronuclei frequencies show substantial variation, there clearly is not any consistent evidence of a bystander effect. GM15036 cells that received ICCM from any dose greater than 0 had frequencies of micronuclei that were similar to each other, and all were lower than the 0-dose values, although the differences were not statistically significant. The frequencies of nucleoplasmic bridges (Figure 2b) showed considerable variation and no consistent dose-related response was seen for either cell line. For both cell lines and for both end-points, i.e. micronuclei and nucleoplasmic bridges, compared to the controls no treatment condition caused any statistically significant change in the frequencies for either of the replicate experiments (p>0.05). These data indicate that neutrons do not induce a bystander effect in these cell lines under these experimental conditions.

### No bystander effect observed due to the photon contamination

**Figure 3:**
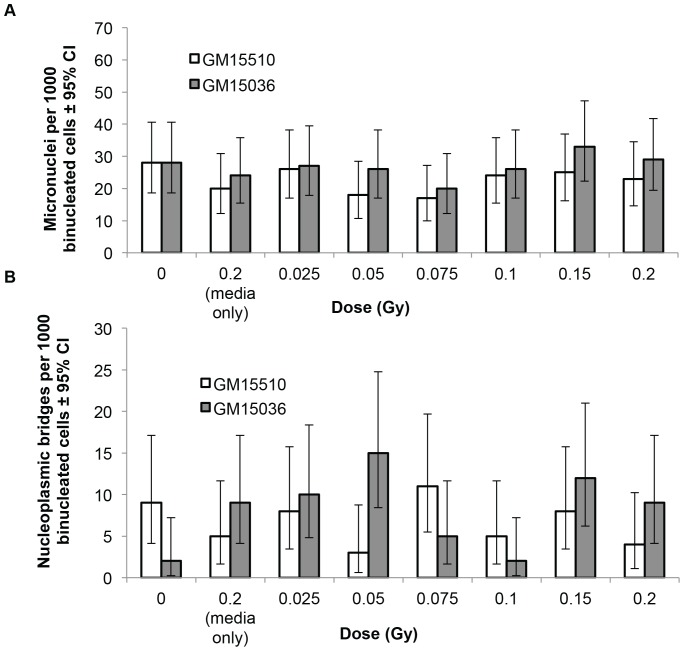
Micronuclei (a) and nucleoplasmic bridges (b) per 1000 binucleated cells in normal human lymphoblastoid cells treated with conditioned media from cells that were directly irradiated with cobalt-60 γ-radiation at doses equivalent to 5% of the neutron doses. Vertical lines represent the 95% confidence intervals.

The neutron beam used in these experiments is contaminated with photons at a level of approximately 5%. Even though a bystander effect was not observed with the neutron exposures, we sought to determine whether photons might cause a bystander effect at the doses employed in these experiments. We cultured GM15510 and GM15036 cells in ICCM obtained from the corresponding cell line that had been irradiated with cobalt-60 at doses equivalent to 5% of the neutron doses. The results, shown in Figure 3, indicate that these low doses of photons did not produce a bystander effect. As a pooled group, cells grown in ICCM showed no significant increase in the frequencies of micronuclei or bridges compared to cells grown in conditioned media from unirradiated cells, as determined by Chi-squared analyses. The media-only control had values similar to the 0 Gy (control) for both endpoints.

### Positive control experiment: Direct damage induced by high doses of cobalt-60 gamma rays

**Figure 4:**
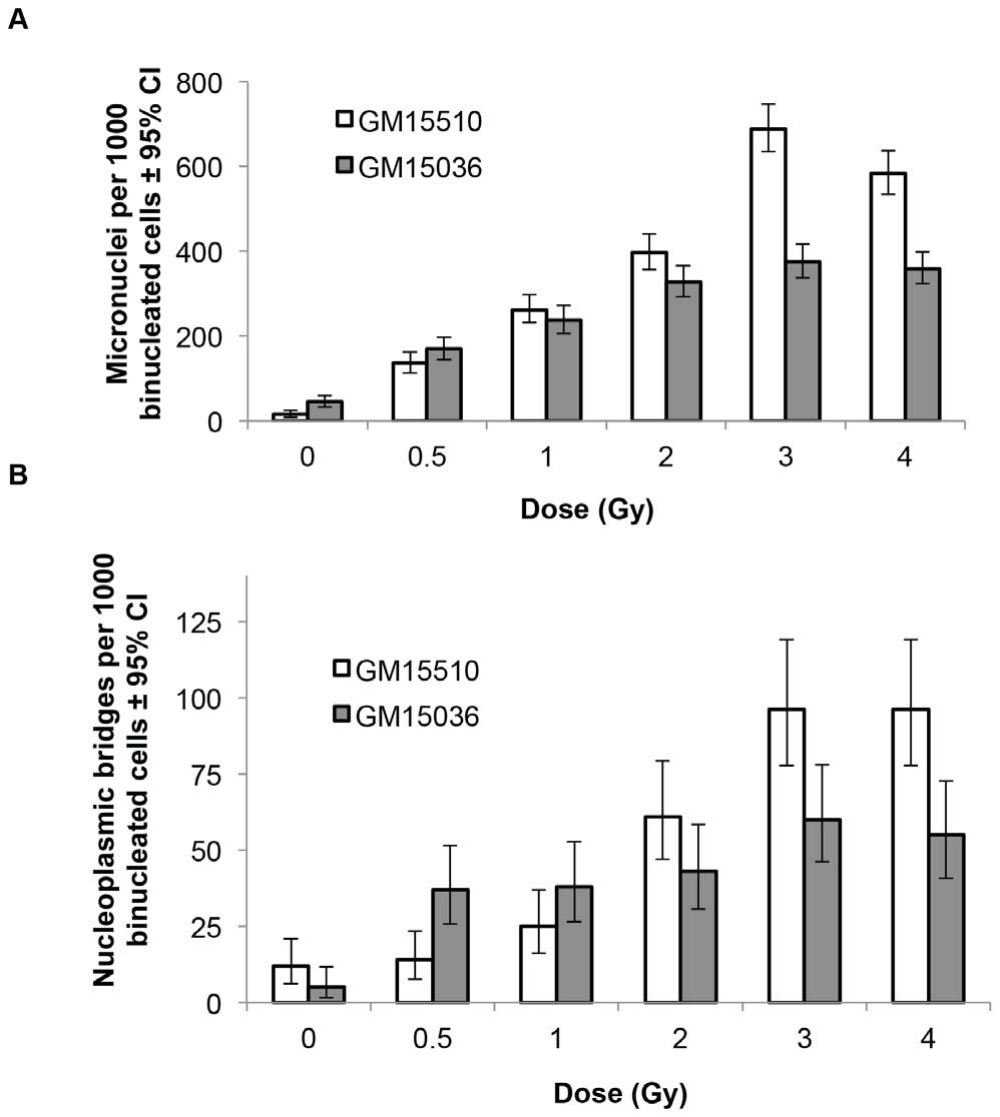
Micronuclei (a) and nucleoplasmic bridges (b) per 1000 binucleated cells in normal human lymphoblastoid cells directly irradiated with high doses of cobalt-60 γ-radiation gamma rays. Vertical lines represent the 95% confidence intervals.

To reaffirm that the experimental conditions used here are capable of seeing a direct (i.e. non-bystander) effect of gamma ray exposure, experiments identical to those performed with neutrons were carried out with high doses of cobalt-60. Direct exposure to these photons with doses from 0 to 4 Gy showed a clear dose responsive increase in the number of micronuclei and the number of bridges per 1000 binucleated cells for both cell lines (Figure 4). The frequencies of micronuclei and bridges appear to saturate at higher doses, indicating that multiple chromosome fragments were packaged in some micronuclei, and that more than one dicentric might have contributed to some bridges. The increases for both micronuclei and bridges with dose were significant with and without considering the 4 Gy data point (p<0.0001).

### Bystander effect induced by high doses of cobalt-60 gamma rays

**Figure 5:**
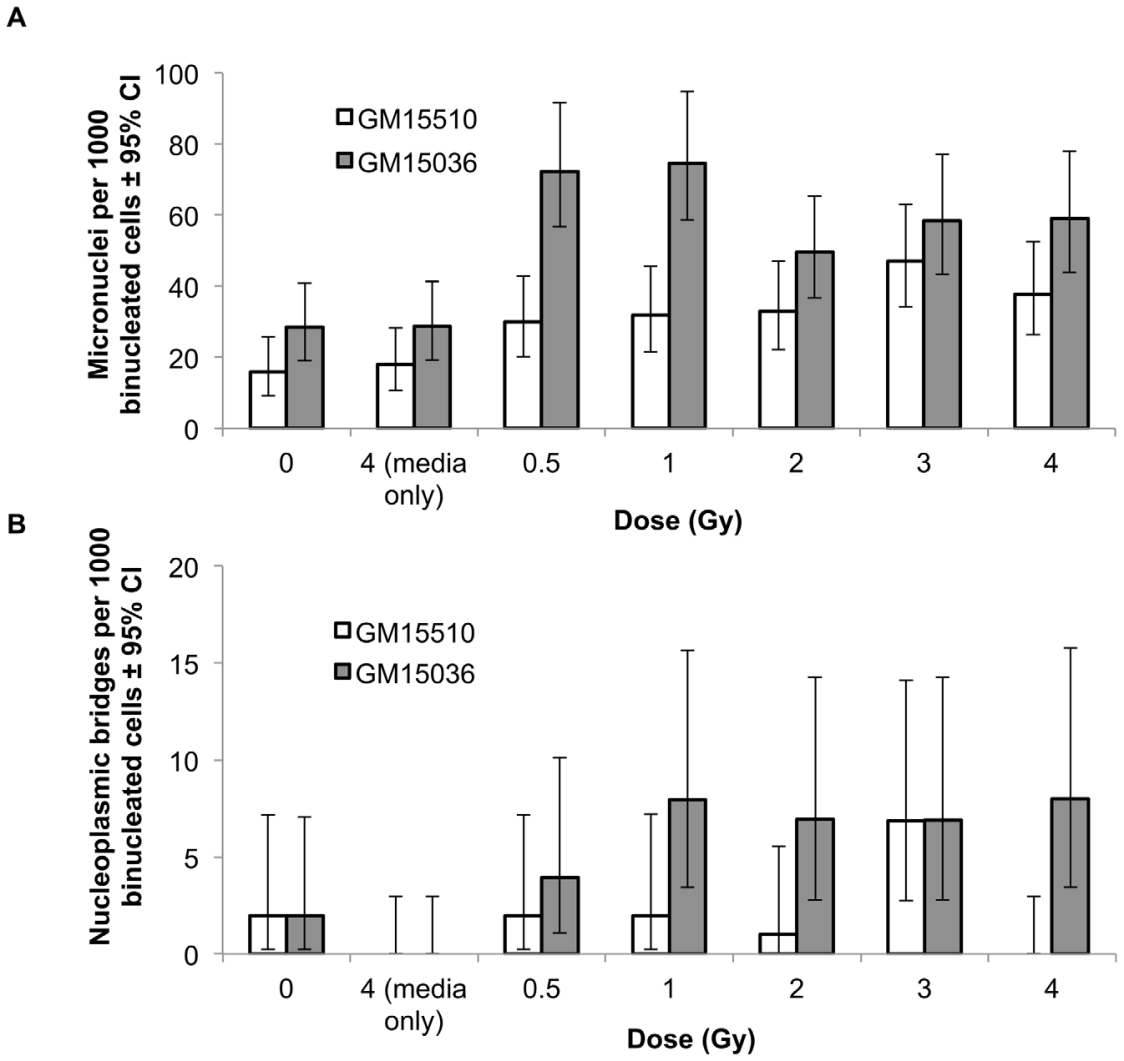
Micronuclei (a) and nucleoplasmic bridges (b) per 1000 binucleated cells in normal human lymphoblastoid cells cultured in conditioned media from cobalt-60 γ-irradiated cells. No bridges were observed in the 4% confidence intervals.

Photons have been known to induce bystander effects in human cells for many years. However, since the neutron exposures and the low-dose photon exposures did not induce a bystander effect, it was important to verify that the cells and the experimental conditions in these experiments, including the serum used in the culture media, are capable of demonstrating a bystander effect if one exists. As expected, GM15510 and GM15036 cells cultured in ICCM obtained from cells exposed to high doses of cobalt-60 gamma rays showed a 2 to 3 fold increase in micronuclei frequencies compared to sham treated controls, clearly indicating induction of a bystander effect (Figure 5a). Compared to cells grown in conditioned media from unirradiated cells, as a pooled group all 6 ICCM cultures for each cell line showed increases in micronuclei (p<0.0001). Nucleoplasmic bridges (Figure 5b) showed a weak bystander effect for GM15510 (p = 0.052), and for GM15036 the effect was highly significant (p<0.0001).

### Bystander component of the total dose response of cobalt-60 gamma rays and neutrons

If the mechanisms of action for direct and non-targeted damage are independent, the total cellular response to ionizing radiation is the sum of the direct and the indirect exposure effects. The bystander components in the total dose response of cobalt-60 gamma rays ranged from approximately 4% to 35% for micronuclei; for bridges, the bystander components ranged up to 6% for GM15510 cells and up to 23% for GM15036 cells (Tables 2 and 3). In contrast, there was no statistically significant bystander component for neutrons (data not shown).

**Table 2:** Percent contribution of the bystander effect to the total direct exposure effect in cobalt-60 irradiated cells for micronuclei.

Cobalt-60 gamma dose (Gy)	Micronuclei/1000 binucleated cells		Micronuclei induced^a^		% bystander component^b^
	Direct exposure	Bystander exposure	Direct exposure	Bystander exposure	
GM15510 cells					
0	15.0	15.9	0.0	0.0	
0.5	136.0	29.9	121.0	14.0	11.6
1.0	262.0	31.9	247.0	16.0	6.5
2.0	396.0	32.9	381.0	17.0	4.5
3.0	688.0	47.0	673.0	31.1	4.6
4.0	583.0	37.8	568.0	21.9	3.9
GM15036 cells					
0	45.0	28.5	0.0	0.0	
0.5	169.0	72.2	124.0	43.7	35.3
1.0	237.0	74.6	192.0	46.2	24.0
2.0	327.0	49.6	282.0	21.1	7.5
3.0	375.0	58.4	330.0	30.0	9.1
4.0	359.0	59.0	314.0	30.5	9.7

a Number of micronuclei per 1000 binucleated cells after subtracting the baseline (0 dose) values for that cell line.

b Percent of the induced total direct exposure response that can be attributed to the induced bystander effect.

**Table 3:** Percent contribution of the bystander effect to the total direct exposure effect in cobalt-60 irradiated cells for nucleoplasmic bridges.

Cobalt-60 gamma dose (Gy)	Bridges/1000 binucleated cells		Bridges induced^a^		% bystander component^b^
	Direct exposure	Bystander exposure	Direct exposure	Bystander exposure	
GM15510 cells					
0	12.0	2.0	0.0	0.0	
0.5	14.0	2.0	2.0	0.0	0.2
1.0	25.0	2.0	13.0	0.0	0.0
2.0	61.0	1.0	49.0	-1.0	−c
3.0	96.0	6.9	84.0	4.9	5.8
4.0	96.0	0.0	84.0	-2.0	−c
GM15036 cells					
0	5.0	2.0	0.0	0.0	
0.5	37.0	4.0	25.0	2.0	7.9
1.0	38.0	8.0	26.0	6.0	23.0
2.0	43.0	6.9	31.0	4.9	16.0
3.0	60.0	6.9	48.0	4.9	10.3
4.0	55.0	8.0	43.0	6.0	14.0

a Number of bridges per 1000 binucleated cells after subtracting the baseline (0 dose) values for that cell line.

b Percent of the induced total direct exposure response that can be attributed to the induced bystander effect.

c Percent bystander component could not be evaluated because the induced bystander effect is negative.

### Relative biological effectiveness (RBE) of the UW CNTS Fast Neutrons

RBE is the ratio of doses that achieve the same biological effect for two radiation types. When the biological responses for both radiation types are linearly related to dose, as seen here, the RBE is also the ratio of the biological effects at the same dose. Here, the neutron RBE was calculated relative to cobalt-60 gamma rays by dividing the frequencies of micronuclei and bridges obtained in cells irradiated directly with neutrons at each dose to the frequencies obtained in cells irradiated directly with photons at the same dose. The RBE of neutrons for all doses and both cell lines is 2.0±0.13 for micronuclei and 5.8±2.9 for bridges (Table 4), indicating that neutrons are 2 to nearly 6 times more effective in damaging these cells compared to cobalt-60 gamma rays.

**Table 4:** Relative biological effectiveness for neutrons relative to cobalt-60 gamma rays for micronuclei and nucleoplasmic bridges.

Dose (in Gy)	Induced^a^ micronuclei/1000 binucleated cells		RBE^b^	Induced^a^ bridges/1000 binucleated cells		RBE^b^
	Cobalt-60 gamma rays	Neutrons		Cobalt-60 gamma rays	Neutrons	
GM15510 cells						
0	0.0	0^c^		0.0	0^c^	
0.5	121.0	255.0	2.1	2.0	40.0	20.0
1.0	247.0	551.4	2.2	13.0	66.9	5.1
2.0	381.0	718.8	1.9	49.0	99.7	2.0
GM15036 cells						
0	0.0	0^c^		0.0	0^c^	
0.5	124.0	173.0	1.4	32.0	32.8	1.0
1.0	192.0	427.9	2.2	33.0	112.0	3.4
2.0	282.0	599.3	2.1	38.0	113.3	3.0
mean +/− S.E. both cell lines			2.0±0.13			5.8±2.9

a The number of micronuclei or bridges per 1000 binucleated cells after subtracting the baseline (0 dose) values, which were 15.0 and 45.0 for micronuclei and 12.0 and 5.0 for bridges for cobalt-60 gamma for GM15510 and GM15036 cells, respectively. For neutrons the baseline values were 28.0 and 29.1 for micronuclei and 2.0 and 11.6 for bridges for GM15510 and GM15036 cells, respectively.

b Relative biological effectiveness: neutrons/cobalt-60 gamma rays.

c Combined values of the controls (pre-radiation, post-radiation and transportation control).

## Discussion

The experiments described here provide no cytogenetic evidence that fast neutrons are capable of inducing a bystander effect through medium-borne factors. The neutron beam used in these experiments was contaminated with photons, necessitating parallel evaluations to determine whether there is a positive or an inhibitory effect of these photons on the results of the neutron experiments. Our observation of an absence of a bystander effect following doses of photons that are associated with exposure to neutrons confirmed that there is a lack of a bystander effect in response to neutrons, regardless of the presence of photons. Wang et al. [56] have suggested that neutrons might suppress gamma ray-induced bystander signaling. They measured apoptosis and cell survival in zebrafish that received bystander signals from fish that were directly irradiated with neutrons. Since the doses of photons that contaminated these neutron exposures exceeded the minimum threshold for inducing a bystander effect [29], [58], Wang et al. [56] suggested that the gamma ray-induced bystander effect might have been suppressed by the neutron exposures. With our data it is difficult to determine whether neutrons have the ability to suppress any bystander effect produced by photons because a source of uncontaminated neutrons is not available. The results shown here suggest that contaminating photons are not a likely confounding factor that interfered with the ability to detect a neutron-induced bystander effect.

Different responses to neutrons were observed in the two cell lines we used. GM15510 cells cultured in ICCM from neutron irradiated cells showed substantial variation in micronuclei frequencies but no consistent dose-related response. In contrast, GM15036 cells had micronuclei frequencies lower than the corresponding 0-dose control value, although the control value was well within the historical range for this cell line. Although we did not measure apoptosis or necrosis, these outcomes may influence the responses seen in these cell lines.

There is clear evidence of a bystander effect in response to high doses of photons when we used the same serum and cell lines as for the neutron experiments. This result indicates that the methods used in our study are capable of detecting a bystander effect if such an effect exists. To the best of our knowledge there is no other factor that could have prevented neutrons from inducing a bystander effect in these cells, assuming a bystander effect even exists. Our findings are in agreement with previous studies that have reported the lack of a bystander effect on neutron exposure using clonogenic cell survival assay in a human skin cell line [29] and zebrafish [56]. However, other studies have reported contrasting results. Watson et al. [59] found that transplantation of a mixture of neutron irradiated and unirradiated bone marrow cells into mice induced instability in the descendants of unirradiated cells as confirmed by measuring chromosomal aberrations, indicating that neutrons induce a bystander effect. However, since the gamma component in neutrons was 25%, it is possible that the observed bystander effect was due to the contaminating photons, which the authors did not rule out. Kinashi et al. [57] studied a neutron-induced bystander effect in boron neutron capture therapy with a cell survival assay as well as cloning and sequencing methods. They reported an increase in the frequency of mutations in the hypoxanthine-guanine phosphoribosyltransferase locus in cells located near the irradiated cells. These results suggest that a neutron bystander effect may be comprised of gene mutations.

The inability of fast neutrons to induce a cytogenetic bystander effect as shown here may be due to different types of damage induced at the molecular level compared to photons. Cellular recognition of DNA damage and the subsequent repair processes may differ between neutrons and photons. Furthermore, due to the lower levels of oxidative damage and free radical production by neutrons compared to photons [60], some of the critical bystander signaling pathways may not be activated. There is also the possibility that a neutron-induced bystander effect, if any, might depend on cell type, the endpoint being evaluated [61], [62], and the energy of the neutrons.

Neutrons, depending on their energy, might be more effective in controlling certain tumor types where conventional photon therapy is ineffective [63] because the oxygen enhancement ratio, i.e. the differential radiosensitivity between poorly oxygenated (more resistant) and well-oxygenated (more sensitive) cells, is reduced with neutrons. Unlike low-LET radiation, for high-LET radiation there is also a reduction in the differential radiosensitivity of cells related to their position in cell cycle [60]. Recently, radiation-induced bystander cells were shown to rescue irradiated cells through intercellular feedback. Chen et al. [40] observed a significant decrease in the number of DNA double-strand breaks, micronuclei frequencies, and the extent of apoptosis in irradiated cells that were co-cultured with unirradiated bystander cells. Observation of an absence of a bystander effect in the present study may help explain the sensitivity of radioresistant tumor cells to neutrons, because there is a possibility that the protection otherwise provided by the bystander effect on the tumor in response to neutrons is absent or not strong enough in magnitude, thereby causing tumor cells to be killed. The risks currently associated with neutron exposure may be over or underestimated depending on which model of risk estimation is used to predict low dose risks from high dose data. Hence, reevaluation of radiation protection standards may be required. The work described in this paper may be relevant for radiation oncologists planning cancer treatments that involve fast neutron or proton radiotherapy, particularly for pediatric patients or pregnant women.

This study used cells that lack gap junctions. There is a possibility that a neutron-induced bystander effect requires physical contact between cells, which could be tested by performing experiments using cell lines such as fibroblasts and keratinocytes that have gap junctions. If no bystander effect is induced in these cell lines, then it may be likely that neutrons do not have any ability to induce a bystander effect. Another possible explanation for the lack of a bystander effect with neutrons observed in this study may be the presence of dimethyl sulfoxide (DMSO), a scavenger of reactive oxygen species [64], [65], which was used to dissolve cytochalasin B that is required for the cytokinesis-block micronucleus assay. Both pre- and post-radiation treatment with DMSO is known to suppress DNA damage in irradiated cells [66]. However, this possibility seems unlikely in the work described here because we observed a bystander effect with an identical procedure involving DMSO when the same cell lines were exposed to photons. However, if a very small bystander effect was in fact induced by neutrons, then it may have been obscured by the DMSO, whereas the bystander effect induced by high levels of photons was too large to be masked by DMSO.

For cells irradiated with high doses of photons, a considerable amount of damage was attributed to the bystander component. The percent contribution by the bystander exposure to the direct exposure was highest at the lowest dose delivered (0.5 Gy) and then it appears to saturate as dose increases, perhaps because there is saturation either of the bystander signals or the cellular responses to those signals [67]. This observation is in agreement with other reports [55], [68], [69]. For cells irradiated with neutrons there is little or no damage that can be attributed to a bystander effect, because as previously noted, there is comparatively less oxidative damage following neutron than gamma exposure.

We report two RBEs for neutron radiation, one for micronuclei and the other for nucleoplasmic bridges. These two genetic endpoints have different mechanisms of formation. Micronuclei are formed from lagging chromosomes and acentric fragments at anaphase, while nucleoplasmic bridges are formed when centromeres of dicentric chromosomes are pulled in opposite directions during mitosis [70]. RBEs of 2.0±0.13 for micronuclei and 5.8±2.9 for nucleoplasmic bridges relative to cobalt-60 suggest that different kinds of genetic damage may be associated with different RBEs. RBE values are known to depend on factors such as linear energy transfer, tissue type, the extent of biological damage, and dose [60]. Knowing the RBE is important for radiation oncologists to determine the dose prescription and the most effective radiotherapy treatment plan for cancer patients. Yang et al. [71] reported RBEs of 2.35 and 2.42 for fast neutrons in immature rat hippocampal cells, as determined by two different cell viability assays. Dagrosa et al. [72] used the cytokinesis-block micronucleus assay and a cell survival assay in a human colon carcinoma cell line and observed an RBE of 4.4 for neutrons in boron neutron capture therapy. RBEs for neutrons as low as 4 to as high as 63 have been reported after measuring life-shortening responses in mice [73], apoptosis [1] and induction of dicentrics [74] in human lymphocytes. These numbers clearly indicate that the RBEs for neutrons vary with the biological system, neutron energy and the end-point. Our RBE values are within the range of what others have reported.

In conclusion, we found no evidence for a bystander effect following exposure to fast neutrons (17 MeV average energy) or to doses of cobalt-60 photons equivalent to 5% of the neutron dose. As expected, a bystander effect was seen with high doses of photons, as evaluated by micronuclei frequencies and nucleoplasmic bridges. These results will facilitate refined estimates of the risk-benefit ratio of neutron therapy and may be valuable to those who are concerned about the health effects of exposure of space travel. We have also shown that these fast neutrons have a relative biological effectiveness of 2.0±0.13 for micronuclei and 5.8±2.9 for bridges compared to cobalt-60. Understanding the biological effects of neutrons may also enable more refined evaluations of the standards for radiation protection and safety.

## Materials and Methods

### Cell lines

Normal human lymphoblastoid cell lines (GM15036 and GM15510) obtained from the Coriell Cell Repository were used for these experiments because they have previously been shown to exhibit a bystander effect in response to gamma radiation [22], [24].

### Cell culture

Cell culture was performed using the standardized protocol provided by Coriell. The serum used in this study was prescreened for its ability to support a bystander effect with the cytokinesis-block micronucleus assay in these same cell lines. Since serotonin has been reported to play a role in the bystander effect [49] and is light sensitive, the bottles containing the culture media were wrapped in aluminum foil and stored in the dark. Cells were cultured and grown in suspension in non-vented T-25 flasks with loosened caps (Corning, NY and ISC BioExpress, Kaysville, UT) containing 10 ml of medium consisting of RPMI1640 (GIBCO, Grand Island, NY or Hyclone, Logan, UT) supplemented with 15% Fetal Bovine Serum (FBS, Atlanta Biologicals, Lawrenceville, GA), 2 mM L-glutamine (GIBCO, Grand Island, NY), penicillin-streptomycin (100 units/ml penicillin G Sodium, 100 µg/ml Streptomycin sulfate in 0.85% saline; GIBCO, Grand Island, NY), fungizone (amphotericin B, 2.5 µg/ml, 0.2 µm filtered; Hyclone, Logan, UT). All cultures were grown and maintained in a fully humidified 5% CO_2_ incubator at 37°C. Approximately every 3 days the cells were counted and passaged by seeding at a concentration of 3×10^5^ cells/ml. Cell culture for all the gamma radiation experiments was performed at Wayne State University (WSU), Detroit, Michigan. For the neutron radiation experiments, cells were grown at WSU up to a concentration of 1×10^6^ cells/mL, then non-vented T-25 flasks were completely filled with media (60 ml) containing cells at this concentration. Two flasks per cell line were then shipped overnight to the University of Washington Medical Center (UWMC), Seattle. Upon arrival, the flasks were immediately placed upright in a fully humidified 5% CO_2_ incubator at 37°C, and their caps were loosened. The flasks were left undisturbed for 24 hours after which the cells were counted and checked for viability using a hemocytometer and trypan blue staining. Cells were then passaged once and cultured as described above.

### Radiation exposures

All neutron irradiations were carried out using the fast clinical neutron therapy system (CNTS) at the University of Washington (Seattle, WA). All gamma irradiations were performed in the Gershenson Radiation Oncology Center, WSU. For neutron irradiations, the cells in culture medium in T-25 flasks were irradiated at room temperature with doses 0 (sham), 0.5, 1, 1.5, 2, 3 and 4 Gy. The CNTS generates fast neutrons by targeting a near monoenergetic 50.5 MeV proton beam at a Be target (10.5 mm thick with a radius of 0.635 cm). The neutron beam is shaped by a primary collimator composed of iron and a secondary collimator made up of individually movable leaves composed of iron with cylindrical polyethylene inserts. Cells in T-25 flasks with culture medium were placed on top of 2 cm of water-equivalent material and irradiated at 0.6 Gy/min from below (gantry at 180 degree) using an open 28.8×28.8 cm^2^ field (SSD of 148 cm). The average energy and neutron mean free path varies with field size and water-equivalent depth (nominal depth of maximum dose is 1.5 cm). For experiments reported in this work, the average neutron energy is about 17 MeV, and the neutron mean free path in water is about 9 cm. For every dose, two flasks were irradiated per cell line: one flask was used to assess the damage induced by direct radiation exposure and the other flask was used for medium transfer for the bystander effect as described below. For the direct damage and the bystander effect experiments, three different controls were used: pre-radiation, post-radiation, and transportation control. Pre-radiation and post-radiation control flasks were sham-irradiated with exposure times corresponding to the lowest (0.5 Gy) and the highest (4 Gy) neutron dose, respectively. The transportation control involved flasks that were transported with the cells that were irradiated, but remained inside the insulated box; these control flasks were further insulated with bubble wrap to maintain their temperature close to 37°C. This box was the same as that used to carry the flasks to and from the laboratory and the radiation center (a 2 minute walk). For the bystander effect an additional media-only control was included; these flasks contained fresh, complete, culture media without any cells and were irradiated at the highest dose, i.e. 4 Gy. Media from these flasks was transferred to non-irradiated cells in the same manner as described for media transfer from flasks that contained cells. Following exposure the flasks were returned immediately to the laboratory and incubated for 28 hours. The cells were then harvested as described below. For each cell line, the neutron bystander experiment was performed twice, once each on different days. Replicate 2 had all the controls as described for replicate 1 except the transportation control was not included.

The Be target system used to generate fast neutrons also delivers a photon dose of about 5% of the neutron dose, which raises the possibility that any bystander effect observed in the experiments could be due to photons rather than to the neutrons. To rule out this possibility, cells were acutely irradiated with cobalt-60 gamma rays at doses equivalent to the 5% of the delivered neutron dose, i.e. 0 (sham), 0.025, 0.05, 0.075, 0.10, 0.15 and 0.20 Gy. We also included a media-only control containing fresh media without cells, which was irradiated at the highest dose, i.e. 0.2 Gy.

As a positive control for the bystander effect, cells were exposed to 0 (sham), 0.5, 1, 2, 3 and 4 Gy cobalt-60 gamma rays. To ensure that the bystander effect observed was actually due to signals produced by the irradiated cells rather than an effect of exposure of the culture medium or an artifact of the media transfer process, a media-only control was included in which fresh media without cells was irradiated at the highest dose (4 Gy) prior to being transferred to non-irradiated cells. For the gamma radiation experiments, flasks were transported to and from the laboratory and the radiation center, a 5-minute car ride, in an insulated container as described above.

### Assessment of direct radiation damage

To assess the effects of direct radiation damage on these cells, Cytochalasin B (6 µg/ml final concentration; Sigma, St. Louis, MO) dissolved in DMSO (Fisher Scientific, Pittsburg, PA) was added four hours after irradiation to directly-irradiated cells to block cytokinesis. The final concentration of DMSO in each culture was 1.1%. The cell cultures were then incubated at 37°C for 28 hours.

### Media transfer

Following irradiation, the cells were left undisturbed until media transfer, which was performed four hours after irradiation as previously described [17]. Briefly, the cell cultures from the non-irradiated and irradiated flasks were transferred to 15 ml centrifuge tubes (Nalgene Nunc International, Rochester, NY) and centrifuged at 370 x g for 5 minutes. To ensure that no cells were transferred along with the media, the supernatant was then passed through 0.22 µm polyethylsulfonate syringe filters (Nalgene). The media from the irradiated cells contains factors secreted by the irradiated cells and hence is considered to be “conditioned”. The media from the non-irradiated (bystander) cells was gently removed by aspiration and replaced with conditioned media. The non-irradiated cells in the conditioned media were then transferred to new T-25 culture flasks and immediately after media transfer 6 µg/ml (final concentration) of Cytochalasin B was added to each culture to block cytokinesis. The cell cultures were then incubated at 37°C for 28 hours.

### Micronucleus Assay - Cell harvesting and slide preparation

Twenty-eight hours following media transfer, the cultures were swirled and pipetted gently to resuspend and break up the clumps of cells. Cells that were directly irradiated as well as those treated with conditioned media were centrifuged onto ethanol-cleaned microscope slides for 4 minutes at 93 x g using a cytocentrifuge (Statspin, Westwood, MA). The slides were air-dried, fixed in 100% methanol (Fisher Scientific Pittsburgh, PA) for 15 minutes, and then stained with 100 µl of acridine orange (0.5 mg/ml in 1X PBS) (Allied Chemical Corporation, Morristown, NJ) for 1 minute in the dark. The excess stain was removed by washing in 1X PBS for 1 minute. The slides were then mounted with 1X PBS and 25×25 mm^2^ glass coverslips.

### Micronuclei scoring criteria

All slides were coded prior to scoring to prevent observer bias. The Nuclear Division Index (NDI) for each dose and treatment was determined by evaluating at least 200 cells and determined according to the following formula: 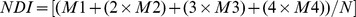
where M1-M4 represent the number of cells with one to four nuclei, respectively, and N is the total number of cells scored [75]. Calculation of the NDI was important to ensure that the number of binucleated cells was sufficient for enumerating micronuclei.

Cells were then evaluated simultaneously for micronuclei, nucleoplasmic bridges and buds according to our “relaxed” criteria [76]. Briefly, only binucleated cells with non-overlapping nuclei were evaluated. Micronuclei were required to be no more than one-third the size of the nuclei, and to be round or oval with smooth edges and stained the same color as the nuclei. Bridges were required to span the entire distance between the two nuclei. Buds were counted only if the stalk was thinner than the widest part of the bud. Since buds did not exhibit a consistent response for either cell line in any of the experiments, we have not included these data in this paper. For each treatment condition, at least 1000 binucleated cells were scored by trained observers. For any experiment, either one observer evaluated all the treatment conditions or the scoring was balanced between two observers such that each evaluated approximately equal numbers of cells for each treatment condition.

### Statistical Analyses

Analysis of variance (ANOVA) was performed to evaluate radiation-induced dose responses in cells directly irradiated with neutrons or high doses of photons, and in cells treated with ICCM from these irradiated cells. For each experiment, the independent variables considered were cell line, dose, and the interaction between cell line and dose. The dependent variables were micronuclei and bridges. The same set of analyses were performed for the neutron bystander data which included replicate experiments as an additional variable. The Tukey HSD test was used for post-hoc evaluations. These analyses were performed using JMP software version 6.0, SAS Institute Inc. Chi squared analyses were used to evaluate changes in the frequencies of micronuclei and bridges in the irradiated cells as a pooled group compared to the unirradiated (0-dose control) cells for the high dose photon experiments, and for the low dose photon contamination experiments.
